# Visual search characteristics in two-way skeet shooters of different performance levels during simulated target viewing

**DOI:** 10.3389/fnins.2026.1783032

**Published:** 2026-04-07

**Authors:** Dongxu Gao, Xindong Ma

**Affiliations:** Division of Sports Science and Physical Education, Tsinghua University, Beijing, China

**Keywords:** eye movements, fixation, gaze, gaze stability, skeet shooting, visual search

## Abstract

**Background:**

Visual search plays a critical role in skeet shooting. Athletes must quickly and rapidly detect, track, and respond to fast-moving targets. Understanding how visual search characteristics differ between shooters of different performance levels during target viewing can provide insight into visuomotor control and visual information processing in dynamic tasks, particularly in skeet shooting.

**Methods:**

A 2 (level of performance: expert, novice) × 2 (target position: high house, low house) × 2 (target type: single target, double target) mixed experimental design was adopted. Differences in eye-movement behavior between expert and novice skeet shooters were analyzed using eye-tracking technology during simulated tasks of target viewing.

**Results:**

The expert group had the lowest reaction time, mean saccade amplitude, number of fixations, pupil diameter, and number of blink compared to the novice counterparts, and the experts had the longest fixation time. There was a variation in fixation and blink count among the target conditions, with significant main effects of target condition. Repeated-measures analysis indicated that there was a significant main effect of group across all the eye-movement and behavioral indicators and no significant group x target condition interaction. Analysis of visualization revealed that highly skilled shooters had more focused gaze patterns, smaller gaze patterns, and more consistent fixation patterns. Conversely, beginner shooters had a more diffuse gaze, more scattered fixation points, and more complicated gaze patterns.

**Conclusion:**

Visual search strategies vary significantly between expert and novice skeet shooters during simulated target-viewing experiments. Compared to amateur shooters, expert shooters exhibit longer fixation times, fewer fixations, smaller saccade amplitudes, less blinking, smaller pupil sizes, and more focused and regular gaze paths. These properties suggest shorter and more efficient visual search behavior, more profound information, and more stable gaze organization during target tracking, reflecting more efficient task-relevant visual information processing.

## 1 Introduction

From the early visual perception and recognition of the clay target, to the visual tracking and visuomotor control, elite athletes process visual information at a very high rate, reaching the functional limits of the vestibulo-ocular tracking system. In skeet shooting, the athletes usually have several milliseconds to perceive and react to important visual data concerning the target flight (Guo Y. et al., 2024). The shooting process has a number of major technical steps, which can generally be defined as looking, staring, starting, driving, aiming, and pulling the trigger. Among them, the most basic and fundamental stage is the one of looking at the target. The shot can only be executed successfully through good tracking of the eye and accurate pointing by the athlete. Thus, effective acquisition and processing of visual information are also key conditions to attain accurate shooting performance (Nascimento et al., 2021).

Visual search in dynamic environments refers to the process by which individuals quickly find, choose, and process information that is relevant to the task under dynamic motor circumstances. In sport, visual information is essential to enable athletes to draw meaningful information about the world, anticipate future events, and produce appropriate motor responses in time. In skeet shooting, this process is particularly challenging due to the high speed, brief visibility, and variable trajectories of the clay targets (Causer et al., 2011; Raffan et al., 2024).

Eye-tracking technology has been utilized extensively in sports-related studies to examine visual information processing. Eye-tracking systems capture several eye-movement cues occurring when performing tasks based on the principles of reflection of the pupil and the cornea, which provide objective information on visual attention and gaze behavior (Kredel et al., 2023). Various experiments involving expert and novice athletes have revealed that expert athletes process visual data more quickly, have more accurate predictions, and are more efficient. These advantages are reflected in higher visual search efficiency and more effective eye-movement strategies (Liu et al., 2020).

In skeet shooting, the previous research has reported that expert shooters have shorter saccade latencies (Morrillo et al., 2006) and their gaze stability is more advanced in the presence of interference conditions (Harris et al., 2024). Research using eye-tracking under live ammunition has also revealed that superior shooters exhibit earlier and prolonged gaze activation compared to sub-elite shooters (Causer et al., 2010). This prolonged stable gaze period allows athletes to program movement parameters more effectively before the execution of the shooting action. Consequently, shooters are able to process information associated with the target trajectory, direction, and velocity more accurately, resulting in more precise responses (Vincze and Jurchiș, 2022).

Despite these findings, significant gaps remain in the current literature. Although expertise-related differences in gaze behavior have been widely documented in shooting and interceptive sports. Relatively little is known about how visual search strategies operate in two-way skeet shooting, which presents a distinct perceptual–motor challenge. Unlike many previously studied interception tasks, targets in two-way skeet may emerge from opposite spatial directions with minimal temporal predictability, which requires rapid allocation of visual attention, anticipatory processing, and continuous updating of spatial predictions. These characteristics impose additional demands on visuospatial processing and gaze stabilization that may amplify expertise-related differences in visual search organization.

Therefore, the present study adopts an expert–novice comparison paradigm to examine eye-movement behavior and visual search characteristics of skeet shooters at different performance levels during simulated target-viewing tasks. By combining multiple eye-movement and behavioral indicators with visualization of gaze distribution and trajectory, this study aims to provide a more comprehensive characterization of visual search organization in a dynamic, directionally uncertain interception task. Because the quality of target observation is closely linked to gun initiation timing and visuomotor coordination in skeet shooting, understanding how visual search differs with expertise may offer insight into perceptual mechanisms underlying skilled performance.

It was hypothesized that shooters of varying performance levels would exhibit different visual search patterns during target viewing. Specifically, expert shooters were expected to demonstrate more efficient visual information processing and visual search strategies, reflected in longer fixation duration, fewer fixations, reduced saccade amplitude, and faster response initiation. These characteristics were expected to support earlier extraction of target flight information, more accurate prediction of target release timing, and more efficient selection of task-relevant visual cues under conditions of directional uncertainty and dynamic target motion.

## 2 Methods

### 2.1 Participants

The participants were grouped into an expert and novice group based on classification criteria documented in earlier studies (Silva et al., 2022). Table 1 summarizes the basic demographic and training characteristics of the participants. The professional sample consisted of ten elite skeet shooters on the national skeet shooting training team, including five males and five females. Their mean age was 23.56 ± 5.11 years, and their mean training experience was 9.41 ± 4.83 years. All expert participants held a competitive level at or above the national athlete standard.

**Table 1:** Demographic, training, and baseline performance characteristics of expert and novice skeet shooters.

Variable	Expert group (*n* = 10)	Novice group (*n* = 10)
Age (years; mean ± SD)	23.56 ± 5.11	19.5 ± 4.21
Sex (male/female)	5/5	6/4
Training experience (years; mean ± SD)	9.41 ± 4.83	2.41 ± 1.53
Competitive level	National-level athletes	Sports school athletes
Average qualifying score (mean ± SD)	119.6 ± 1.65	116.5 ± 1.43

The novice group included 10 s-level two-way skeet shooters of the Beijing Shooting Sports School, comprising of six males and four females. The mean age of the novice group was 19.53 ± 4.21 years, with a mean training experience of 2.41 ± 1.53 years. All novice participants had participated in domestic competitions, and their average qualifying scores in the semi-annual competition was 105 or more.

All the participants were right-handed, with either normal or corrected-to-normal vision. In addition to handedness, ocular dominance was assessed for each participant using the Miles test. All participants demonstrated right-eye dominance, which was consistent with their right-handed shooting posture. Because both expert and novice groups showed homogeneous handedness and dominant-eye distribution, these variables were not included as independent factors in the statistical analysis. However, their potential influence on visual search behavior, particularly under different target flight directions (high-house vs. low-house), was considered during interpretation of the results. Because the study involved elite national-level skeet shooters, participant availability was limited, resulting in a relatively small sample size (*n* = 10 per group). Similar sample sizes have been reported in previous eye-tracking studies involving elite athletes where recruitment of highly trained participants is inherently constrained. No formal *a priori* power analysis was conducted prior to data collection because participant recruitment was determined by the availability of athletes within the national training program. However, effect sizes were calculated for all between-group comparisons to provide an estimate of the magnitude of the observed differences and to support interpretation of the statistical results. They were informed about the experimental procedures, and provided written informed consent before the participation. Ethical approval for this study was obtained from Chinese Institute of Sport Science Ethics Committee (CISSEC; approval number 2024.09.02). The study was conducted in accordance with the Declaration of Helsinki.

### 2.2 Experimental design and instrumentation

The mixed experimental design of 2 (level of performance: expert, novice) × 2 (target position: high house, low house) × 2 (target type: single target, double target) was used in this study. Since two-way skeet shooting consists of four common target-throwing conditions, this design allowed comprehensive coverage of the main shooting scenarios and enabled systematic examination of visual search characteristics in skeet shooters at different performance levels during simulated target viewing. The between-subject factor was performance level, whereas target position and target type were considered within-subject factors. The behavioral and eye-movement indicators of shooters at various levels were compared in all the target-viewing conditions.

**Figure 1:**
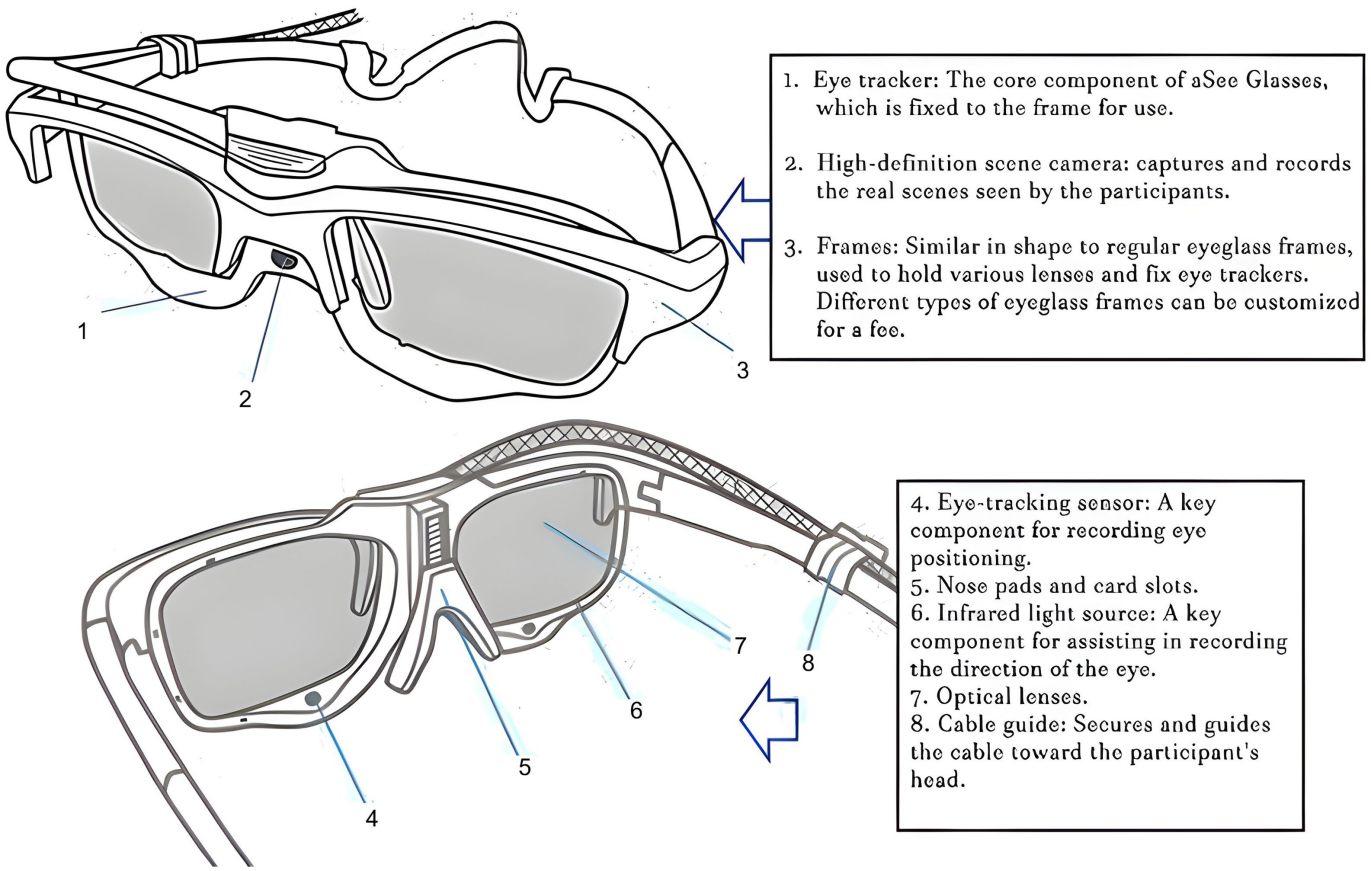
The aSee Glasses eye-tracking system used for recording eye-movement data during the simulated skeet-shooting task.

The aSee Glasses eye-tracking system was used as an eye-movement instrument (Beijing Qixin Yiwei Information Technology Co., Ltd., China). Figure 1 shows the structure and components of this eye tracking system. The system had a horizontal tracking range of up to 80° and a vertical tracking range of up to 60°, with a gaze accuracy of approximately 0.5°. Such accuracy levels are comparable to those reported for wearable eye-tracking systems commonly used in sports and behavioral research (Kredel et al., 2023). Eye movements were recorded, and gaze-related images were captured by a three dimensional eye tracking algorithm (Gao D. et al., 2025). The videos were shown on a large screen monitor that was attached to a Dell laptop computer. Eye-movement recordings were synchronized with video stimuli, and the data were stored in the aSee Glasses Studio software for subsequent processing and analysis.

### 2.3 Experimental materials

The study employed a laboratory-based simulation approach using video recordings of clay target flight trajectories. The video stimulus was filmed at the National Skeet Shooting Training Site, Putian using a SONY AX700 camera in manual high-definition mode. Shooters in the median height range were chosen based on the height statistics of the team to be representative. The camera was raised to the height of 1.6 m, corresponding to the shooter’s eye level, and was mounted at the center of Station 4 so as to view the target flight in the perspective of the shooter.

**Figure 2:**
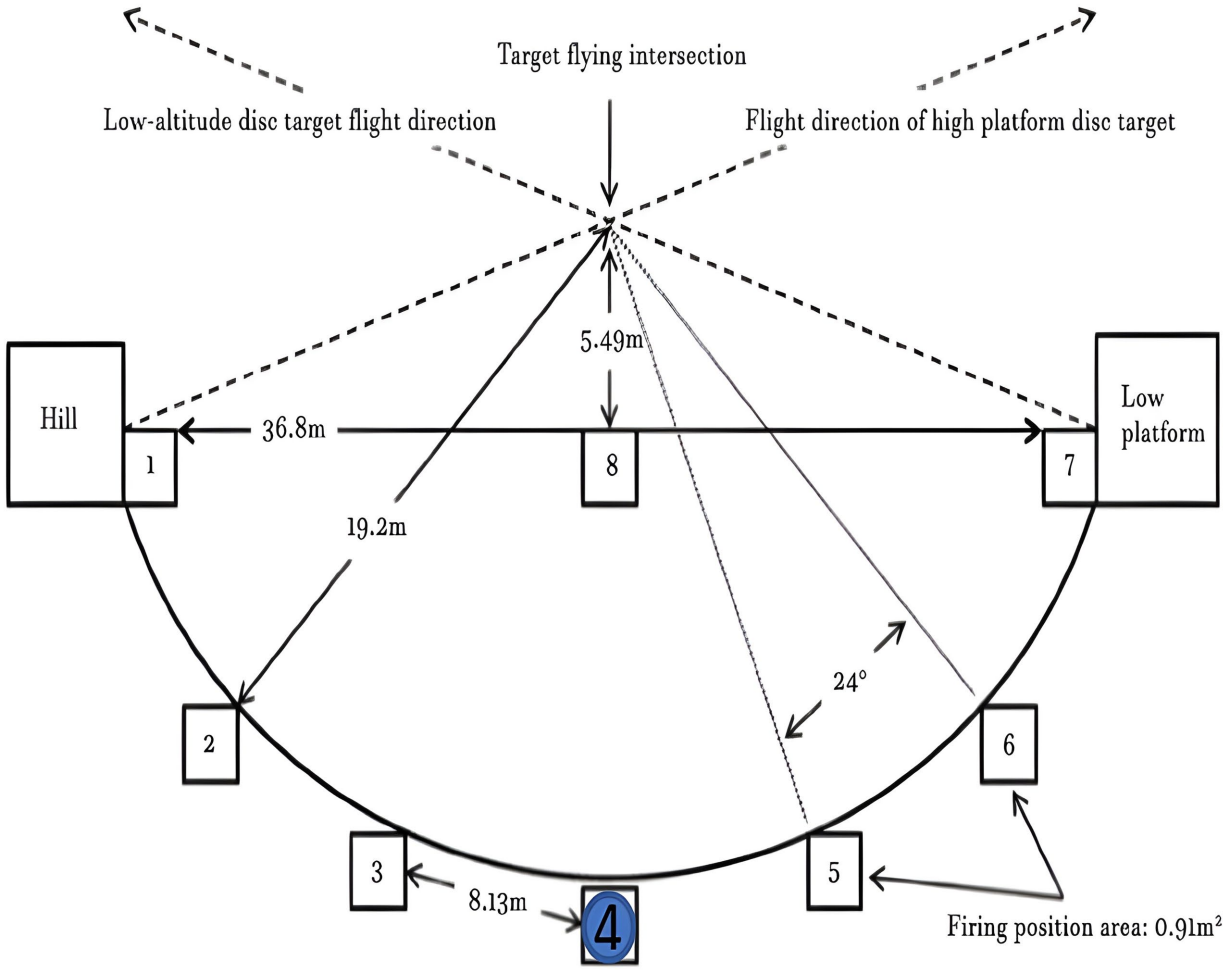
Schematic illustration of the stimulus acquisition setup at the skeet shooting range to record video stimuli.

The video recordings were done at 3:00 p.m. to 5:00 p.m. when the daylight conditions were steady and the glare was minimal. The speed of the wind was not more than level 3 at the time of recording, which guarantees the stable and reproducible target trajectories. This setup allowed the recorded video stimuli to closely match visual perspective and viewing experience encountered by the athletes during real skeet shooting. The stimulus acquisition layout is shown in Figure 2.

During recording, the athletes observed the target release in real time to confirm that the visual perspective of the videos was consistent with actual target viewing. Twenty (20) video clips were first captured in each of the four target-viewing conditions (i.e., high house single target, low house single target, high house double target, and low house double target). After expert screening, fifteen (15) high-quality video clips in each condition were selected and videos with visual interference or abnormal target movement were excluded to ensure consistency with real shooting conditions.

The location of Station 4 was chosen as the site of stimulus acquisition because it was the point of the fan-shaped shooting arc and offers an extensive and representative target flight trajectory. This position facilitates visual tracking and highlights differences in visual search behavior. This made it suitable to examine visual characteristics associated with different performance levels.

### 2.4 Experimental procedure

The experiment was conducted in well-lit, independent room situated behind shooting range. The participants were instructed to stand about 1.5 m facing large-screen monitor. The height of the display was adjusted so that the center of the screen was aligned with the participant’s line of sight, thereby simulating a natural target-viewing angle. The eye-tracking device was fitted on the participants and their comfort was put in the first place during the session. The pupil position was then calibrated using the aSee Glasses system in a three-point calibration procedure.

Prior to the start of experiment, participants were provided with standardized instructions which explains the experimental content and procedures. After confirming their understanding and consent, two sets of practice videos were presented to allow participants to adapt to the experimental environment and become familiar with the task. In the experiment, each of the participants was exposed to a total of 60 video clips of clay target flight in random order, 15 video clips per target condition. The participants were told to watch the target flight and use the space bar when the clay target was visible. In case no response was provided in time, before the conclusion of a video, the trial proceeded to the next video.

**Figure 3:**
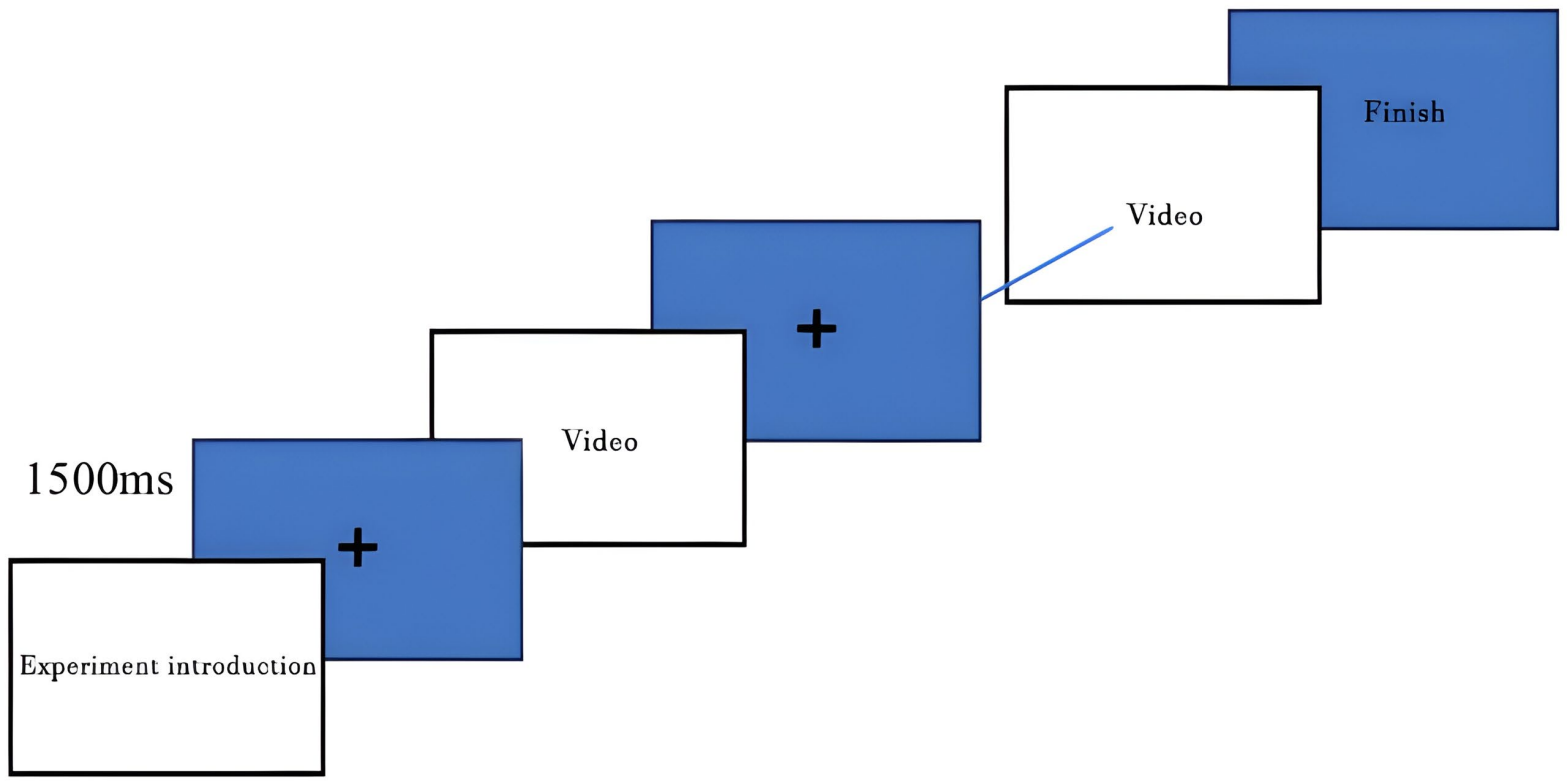
Trial sequence of the simulated target-viewing task, including instruction display, fixation cross, video presentation, response period, and a 1,500 ms inter-trial interval.

**Figure 4:**
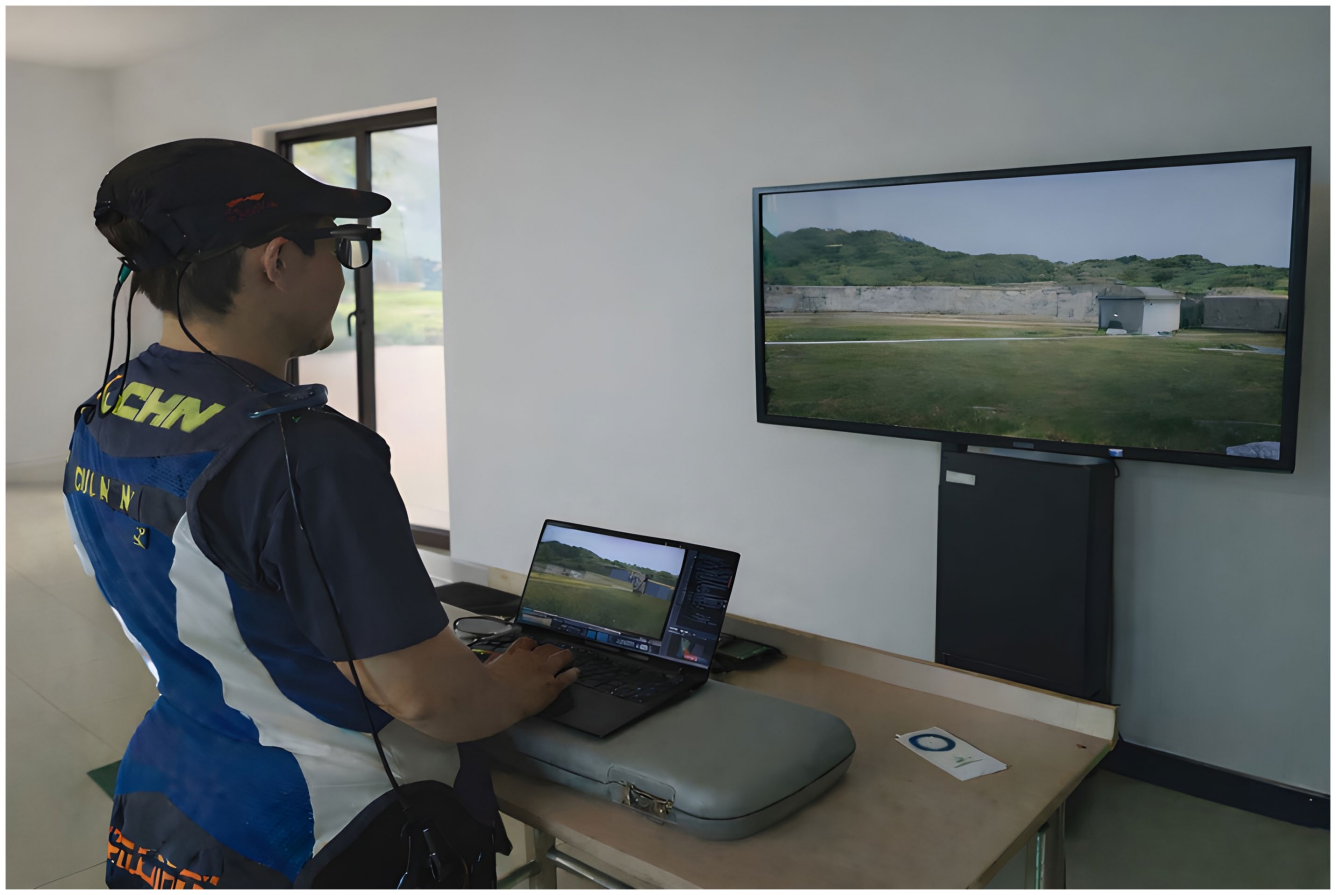
Experimental setup for the simulated skeet-shooting task.

A 1500 ms inter-trial interval occurred after each of the trials to enable the participants to relax and prepare to receive the next stimulus. Before every trial, a fixation cross (“+”) was shown at the center of the screen to make sure that gaze was initiated in the optimal recording range of the central visual field. Figure 3 shows the sequence of events in each trial, such as display of instructions, fixation cross, and display of video, response period and inter-trial interval. As Figure 4 is depicting the entire experimental configuration, positioning of the participants and equipment.

The Key Event feature of the aSee Glasses Studio software was used to capture the behavioral responses. Reaction time was obtained from the raw data and defined as the interval between target appearance and the participant’s key press. The participants were told to estimate the release of the targets and as soon as possible to react to the appearance of clay targets. The software automatically logged the time every key was pressed and synchronized it with the eye-movement data.

Since the video trials were quite numerous, efforts were put to keep the participants engaged and focused during the experiment. Prior to testing, participants were informed that they would be asked after the experiment to recall and describe details of the target flight videos they had observed, including how they visually tracked the target and perceived its trajectory. This procedure was used to encourage sustained attention and ensure consistent engagement during the target-viewing task.

### 2.5 Description of test indicators

The eye-movement and behavioral indicators in this study were selected on basis of previous studies (Silva et al., 2022; Gao et al., 2024). These indicators were used to characterize visual search behavior during simulated target viewing. The definitions and interpretations of these indicators have been summarized in Table 2.

**Table 2:** Definitions and interpretation of eye-movement and behavioral indicators used to assess visual search behavior.

Indicator	Definition	Unit	Interpretation
Reaction time	Time interval between the appearance of the clay target and the participant’s key response	ms	Shorter reaction time indicates faster visual processing and response initiation
Mean saccade amplitude	Average angular distance of saccadic eye movements during target viewing	Degrees (°)	Smaller amplitudes reflect more focused and efficient visual search
Fixation count	Total count of fixations recorded during target viewing	Numbers	Fewer fixations indicate more efficient extraction of task-relevant visual information
Fixation duration	Average duration of individual fixations during target viewing	s	Longer durations reflect sustained attention on relevant visual regions
Pupil diameter	Average pupil size recorded during target viewing	mm	Larger diameter indicates higher cognitive load or attentional demand
Blink count	Total number of eye blinks during target viewing	Numbers	Fewer blinks indicate more stable visual attention and reduced interruption of visual input

All indicators were derived from eye-tracking data recorded during simulated target viewing tasks.

Fixation count shows the efficiency of processing of visual information during viewing of target. Fewer fixations demonstrate more efficient extraction of task-relevant visual information. Fixation duration represents depth of information processing, with longer durations which indicate sustained attention to relevant visual regions. In the present study, fixation was defined based on gaze stability relative to the visual scene using the velocity-threshold identification (I-VT) algorithm implemented in the aSee Glasses system. Because the clay target was continuously moving, fixation events included both short periods of stable gaze on predicted target-trajectory regions and brief gaze stabilization during smooth pursuit of the moving target. Therefore, fixation duration in this study reflects sustained visual engagement with task-relevant spatial regions rather than strictly stationary gaze on a fixed location. This operational definition is consistent with previous eye-tracking research in dynamic visuomotor tasks, where fixation and pursuit are functionally integrated during continuous target tracking. Pupil diameter is commonly used as an indicator of cognitive load and attentional resource allocation. The larger diameters reflects increased cognitive demand. Blink count reflects physiological and attentional stability during task performance; while, fewer blinks indicates reduced interruption of visual input under visually demanding conditions.

Mean saccade amplitude reflects the spatial extent of visual search and the efficiency of information extraction during tracking of clay targets. Smaller saccade amplitudes indicate more focused visual search behavior and stronger visual tracking stability. Reaction time was used as a behavioral indicator of prediction and response speed during target viewing. The shorter reaction times indicate faster perceptual processing and response initiation.

In addition to quantitative indicators, gaze trajectory and visual attention heat maps were generated to visualize eye-movement behavior. Gaze trajectory maps were constructed by connecting fixation locations in temporal order, which illustrate the spatial distribution and sequence of eye movements, allowing evaluation of visual tracking strategies. Heat maps were generated based on fixation dwell duration with the purpose of visually representing areas of concentrated visual attention, supporting interpretation of visual search patterns (Li et al., 2023; Podladchikova et al., 2009; Krejtz et al., 2018).

The aSee Glasses system recorded both eye-position data and synchronized world-view video, allowing gaze coordinates to be projected onto the visual scene. In the present study, gaze trajectory visualization and heat maps were generated based on scene-referenced gaze mapping to qualitatively illustrate spatial distribution of visual attention during target viewing. However, because the primary aim was to compare global visual search characteristics between skill levels, quantitative analysis focused on established eye-movement metrics (fixation, saccade, pupil, and blink indicators). Continuous gaze–target positional coupling was not analyzed as a separate variable, which is acknowledged as a methodological limitation and a potential direction for future research.

Eye-movement data were recorded at a sampling rate of 60 Hz using the aSee Glasses system. This sampling frequency is commonly used in mobile eye-tracking studies investigating visual search behavior in sports contexts and is sufficient for detecting fixation patterns and general saccadic characteristics during dynamic viewing tasks (Kredel et al., 2023). Fixations and saccades were classified using the velocity-threshold identification (I-VT) algorithm implemented in the system software, with a velocity threshold of 30°/s to distinguish saccadic from stable gaze periods. Blink events and missing samples were automatically detected based on the temporary loss of pupil signal and were excluded from fixation and saccade calculations. Trials were excluded if gaze tracking loss exceeded 20% of the recording duration or if calibration error exceeded 1°. Overall data quality was high and comparable across groups, with valid gaze data exceeding 94% on average across participants and conditions. No systematic differences in data loss were observed between expert and novice groups, and identical preprocessing procedures were applied to all recordings.

### 2.6 Statistical analysis

Eye-movement and behavioral data recorded by the eye-tracking system were exported and organized using Microsoft Excel. Descriptive statistics were calculated for all eye-movement and behavioral indicators and are reported as mean ± standard deviation. Between-group differences under each target-viewing condition were examined using independent-samples *t*-tests. To further examine the effects of performance level and target condition, repeated measures ANOVA was performed and Greenhouse–Geisser method was applied, where the assumption of sphericity was violated. Type III sums of squares were used to calculate F statistics. Homogeneity of variance between the expert and novice groups was assessed using Levene’s test. Statistical analyses were performed using SPSS version 26.0 (IBM, USA). *p* < 0.05 was considered as the level of statistical significance.

For condition-wise group comparisons, independent-samples *t*-tests were conducted. When the assumption of homogeneity of variance was violated according to Levene’s test (*p* < 0.05), Welch’s *t*-test was used. All tests were two-tailed. Effect sizes were estimated using Cohen’s d for between-group comparisons, and these values were interpreted alongside significance tests to provide additional information about the magnitude of observed differences. Given the relatively small sample size, effect size estimates were interpreted cautiously as they may be sensitive to sampling variability. Because multiple condition-wise comparisons were exploratory in nature, results were interpreted cautiously with emphasis on effect patterns rather than isolated significance values. The primary inferential framework of the study was the mixed-design repeated-measures ANOVA, which evaluated main effects of group and target condition and their interaction. Statistical assumptions and decision rules were applied consistently across all analyses.

## 3 Results

### 3.1 Overall results of eye-movement tests

Descriptive statistics for reaction time, mean saccade amplitude, fixation count, pupil diameter, fixation duration, and blink count among all target-viewing conditions are presented in Table 3. Among all conditions, expert shooters consistently exhibited significantly shorter reaction times than those of novice shooters. For instance, reaction time in the high house single-target condition was observed as 4544.81 ± 233.92 ms for experts compared with 4808.43 ± 40.76 ms for novices (*p* < 0.05).

**Table 3:** Descriptive statistics and statistical comparison of eye-movement and behavioral indicators across target-viewing conditions (mean ± SD).

Sr. No.	Indicator	Condition	Expert (*M* ± SD)	Novice (*M* ± SD)	Variance homogeneity (Levene’s P)	Cohen’s d	Statistical Test
1	Reaction time (ms)	High house single	4544.81 ± 233.92ᵃ	4808.43 ± 40.76ᵇ	<0.001	−1.57	Welch *t*-test
		Low house single	4541.37 ± 172.09ᵃ	4833.84 ± 54.46ᵇ	0.051	−2.29	Independent *t*-test
		High house double	4565.90 ± 185.13ᵃ	4835.49 ± 48.60ᵇ	0.615	−1.99	Independent *t-*test
		Low house double	4533.85 ± 230.31ᵃ	4824.04 ± 52.69ᵇ	0.001	−1.74	Welch *t*-test
2	Mean saccade amplitude (°)	High house single	188.05 ± 19.48ᵃ	250.97 ± 14.22ᵇ	0.482	−3.69	Independent *t*-test
		Low house single	185.89 ± 17.52ᵃ	246.04 ± 13.62ᵇ	0.523	−3.83	Independent *t*-test
		High house double	188.95 ± 22.36ᵃ	250.02 ± 12.31ᵇ	0.466	−3.34	Independent *t*-test
		Low house double	189.76 ± 20.58ᵃ	248.69 ± 12.75ᵇ	0.153	−3.43	Independent *t*-test
3	Fixations count (n)	High house single	2.73 ± 0.43ᵃ	3.97 ± 0.25ᵇ	0.074	−3.53	Independent *t*-test
		Low house single	2.83 ± 0.42ᵃ	4.07 ± 0.40ᵇ	0.523	−3.02	Independent *t*-test
		High house double	6.86 ± 0.30ᵃ	8.11 ± 0.41ᵇ	0.466	−3.46	Independent *t*-test
		Low house double	6.75 ± 0.29ᵃ	7.83 ± 0.27ᵇ	0.864	−3.83	Independent *t*-test
4	Pupil diameter (mm)	High house single	2.42 ± 0.28ᵃ	3.47 ± 0.06ᵇ	<0.001	−5.19	Welch *t*-test
		Low house single	2.53 ± 0.21ᵃ	3.50 ± 0.28ᵇ	0.348	−3.87	Independent *t*-test
		High house double	2.54 ± 0.19ᵃ	3.59 ± 0.29ᵇ	0.186	−4.27	Independent *t*-test
		Low house double	2.52 ± 0.22ᵃ	3.55 ± 0.26ᵇ	0.153	−4.21	Independent *t*-test
5	Fixation duration (s)	High house single	1.29 ± 0.13ᵃ	1.17 ± 0.09ᵇ	0.073	1.06	Independent *t*-test
		Low house single	1.32 ± 0.12ᵃ	1.21 ± 0.08ᵇ	0.233	1.10	Independent *t*-test
		High house double	1.38 ± 0.13ᵃ	1.20 ± 0.05ᵇ	0.001	1.84	Welch *t*-test
		Low house double	1.35 ± 0.14ᵃ	1.21 ± 0.07ᵇ	0.012	1.26	Welch *t*-test
6	Blink Count (n)	High house single	0.81 ± 0.15ᵃ	1.40 ± 0.16ᵇ	0.608	−3.80	Independent *t*-test
		Low house single	0.88 ± 0.21ᵃ	1.41 ± 0.16ᵇ	0.733	−2.84	Independent *t*-test
		High house double	1.09 ± 0.33ᵃ	1.87 ± 0.27ᵇ	0.202	−2.59	Independent *t*-test
		Low house double	1.01 ± 0.21ᵃ	1.87 ± 0.13ᵇ	0.148	−4.92	Independent *t*-test

ᵃ^,^ᵇ Different superscript letters indicate significant differences between expert and novice groups within the same condition (*p* < 0.05). Homogeneity of variance was assessed using Levene’s test. Welch’s *t*-test was applied when variance homogeneity was violated. Cohen’s d was computed using pooled standard deviation and is reported as d.

Mean saccade amplitude was found significantly lower in expert shooters than that in novices across all conditions (e.g., high house single: 188.05 ± 19.48° vs. 250.97 ± 14.22°, *p* < 0.05). Similarly, Expert group showed significantly reduced fixation count in all conditions as compared to the novice group (e.g., high house single: 2.73 ± 0.43 vs. 3.97 ± 0.25, *p* < 0.05). Pupil diameter was found significantly smaller in experts in all conditions (e.g., high house single: 2.42 ± 0.28 mm vs. 3.47 ± 0.06 mm, *p* < 0.05), and blink count was also lower in experts as compared to novice (e.g., high house single: 0.81 ± 0.15 vs. 1.40 ± 0.16, p < 0.05).

In contrast, fixation duration was significantly longer in experts than in novices, particularly during double-target conditions (e.g., high house double: 1.38 ± 0.13 s vs. 1.20 ± 0.05 s, *p* < 0.05). Significance between-group differences within each condition are indicated by different superscript letters in Table 3.

Homogeneity of variance between the expert and novice groups was assessed using Levene’s test (Table 3). In the high house single-target condition, reaction time and pupil diameter showed heterogeneity of variance between groups, whereas mean saccade amplitude, fixation count, fixation duration, and blink count did not show heterogeneity. In the low house single-target condition, all measured indicators met the assumption of variance homogeneity. In the high house double-target condition, heterogeneity of variance was observed for mean saccade amplitude and fixation duration, while reaction time, fixation count, pupil diameter, and blink count showed homogeneity. In the low house double-target condition, reaction time and fixation duration exhibited heterogeneous variance, whereas mean saccade amplitude, fixation count, pupil diameter, and blink count showed the homogeneity assumption.

### 3.2 Effects of group and target condition

Results of the repeated-measures ANOVA are summarized in Table 4. A significant main effect of group was observed for reaction time (*F* = 68.637, *p* < 0.001), mean saccade amplitude (*F* = 255.626, *p* < 0.001), fixation count (*F* = 234.528, *p* < 0.001), pupil diameter (*F* = 384.050, *p* < 0.001), fixation duration (*F* = 33.968, *p* < 0.001), and blink count (*F* = 205.453, *p* < 0.001).

**Table 4:** Results of repeated-measures ANOVA examining the effects of group and target condition on eye-movement and behavioral indicators.

Indicator	Source	Type III sum of squares	df	*F*	*p*
Reaction time (ms)	Group	1,556,466.53	1	68.637	<0.001
	Target condition	7,125.03	3	0.105	0.957
	Group × target condition	3,157.89	3	0.046	0.987
Mean saccade amplitude (°)	Group	73,857.73	1	255.626	<0.001
	Target condition	179.09	3	0.207	0.892
	Group × target condition	42.53	3	0.049	0.986
Fixations count (n)	Group	28.96	1	234.528	<0.001
	Target condition	318.36	3	859.317	<0.001
	Group × target condition	0.10	3	0.274	0.844
Pupil diameter (mm)	Group	21.04	1	384.050	<0.001
	Target condition	0.15	3	0.915	0.438
	Group × target condition	0.02	3	0.131	0.941
Fixation duration (s)	Group	0.389	1	33.968	<0.001
	Target condition	0.038	3	1.117	0.348
	Group × target condition	0.013	3	0.384	0.765
Blink Count (n)	Group	9.524	1	205.453	<0.001
	Target condition	2.344	3	16.855	<0.001
	Group × target condition	0.365	3	2.623	0.057

Repeated-measures analysis of variance was conducted with group (expert vs novice) as a between-subject factor and target condition (high house single, low house single, high house double, low house double) as a within-subject factor. *p* < 0.05 was considered statistically significant.

The main effect of the target condition was significant for fixation count (*F* = 859.317, *p* < 0.001) and blink count (*F* = 16.855, *p* < 0.001), whereas no significant effects were detected for reaction time, mean saccade amplitude, pupil diameter, or fixation duration (*p* > 0.05). No significant group × target condition interaction was observed for any indicator (*p* > 0.05).

To further clarify the variation across target-viewing conditions, within-group comparisons were conducted separately for the expert and novice groups. In the expert group, fixation count and blink count showed significant variation across target conditions, with higher values observed during double-target trials compared with single-target trials (*p* < 0.05). It indicated the increased visual sampling demands under more complex viewing conditions. Other indicators, including reaction time, mean saccade amplitude, pupil diameter, and fixation duration, did not differ significantly across conditions (*p* > 0.05). It suggested stable and consistent visual search strategies in expert shooters regardless of target configuration.

**Figure 5:**
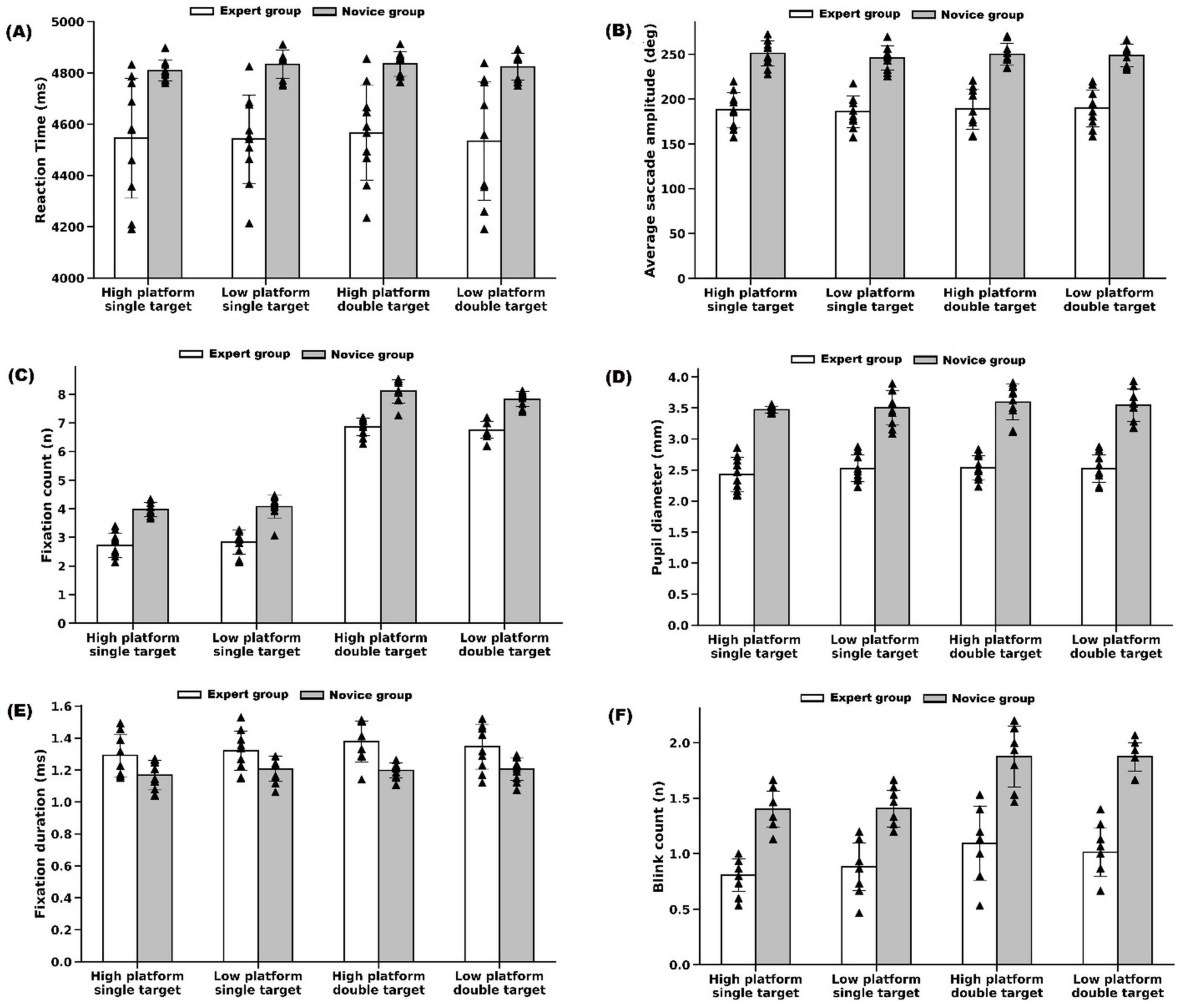
Comparison of eye-movement and behavioral indicators between expert and novice shooters across target-viewing conditions. **(A)** Reaction time (ms). **(B)** Mean saccade amplitude (°) **(C)** Fixation count (n). **(D)** Pupil diameter (mm). **(E)** Fixation duration (s) **(F)** Blink count (n). Data are presented across four target-viewing conditions: high house single target, low house single target, high house double target, and low house double target. Error bars represent standard deviation, and arrowheads indicate individual participant data point.

In contrast, the novice group demonstrated greater variability across target conditions. Fixation count and blink count increased significantly during double-target conditions compared with single-target conditions (*p* < 0.05), reflecting increased visual instability and attentional demand under higher task complexity. However, reaction time, mean saccade amplitude, pupil diameter, and fixation duration showed no statistically significant differences across conditions (*p* > 0.05). These findings indicate that although both groups were influenced by task complexity, novice shooters exhibited less stable and less efficient visual search behavior across varying target-viewing scenarios (see Figure 5).

### 3.3 Visualization of eye-movement indicators

#### 3.3.1 Visual attention heat maps

**Figure 6:**
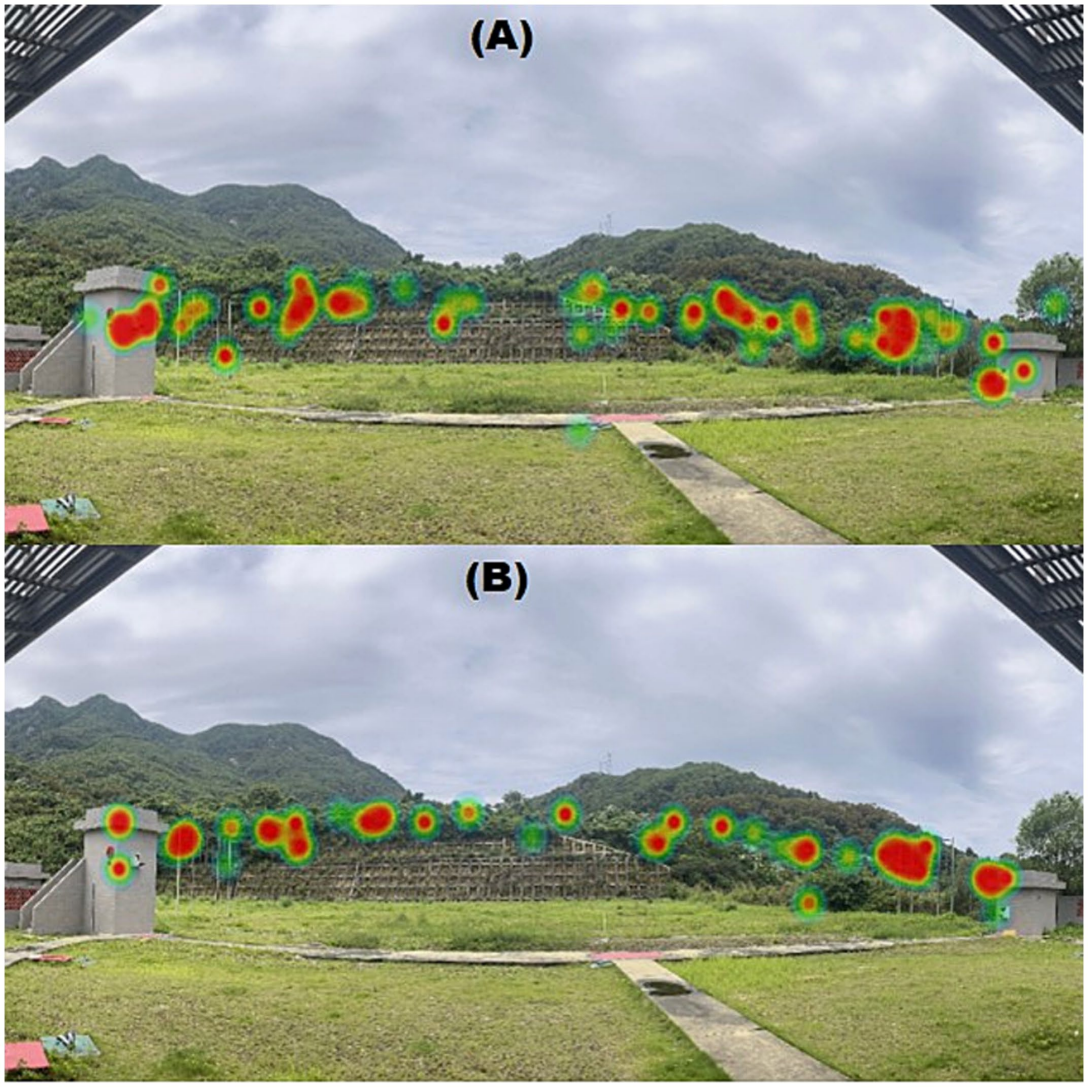
Representative visual attention heat maps for novice **(A)** and expert shooters **(B)** during target viewing. Warmer colors indicate regions with higher fixation density.

Representative visual attention heat maps for novice and expert shooters are shown in Figures 6A,B, respectively. Fixation duration is represented by a color gradient (red > yellow > green), while areas without color indicate regions not viewed by participants. The expert group demonstrated a more concentrated and coherent fixation distribution, whereas the novice group exhibited a wider and more dispersed gaze pattern. Compared with novices, experts exhibited longer mean fixation durations and fewer fixation points during target viewing, as exhibited by the numerical values given in Table 3 (e.g., high house double-target condition: 1.38 ± 0.13 s in experts vs. 1.20 ± 0.05 s in novices; 2.73 ± 0.43 vs. 3.97 ± 0.25 fixations, respectively).

#### 3.3.2 Gaze trajectories

Gaze trajectories were constructed by connecting fixation points in temporal order. Each fixation point represents a gaze location, and the size of the circle indicates fixation duration, with larger circles corresponding to longer gaze time at that location. This visualization reflects the spatial and temporal organization of eye movements during target viewing.

**Figure 7:**
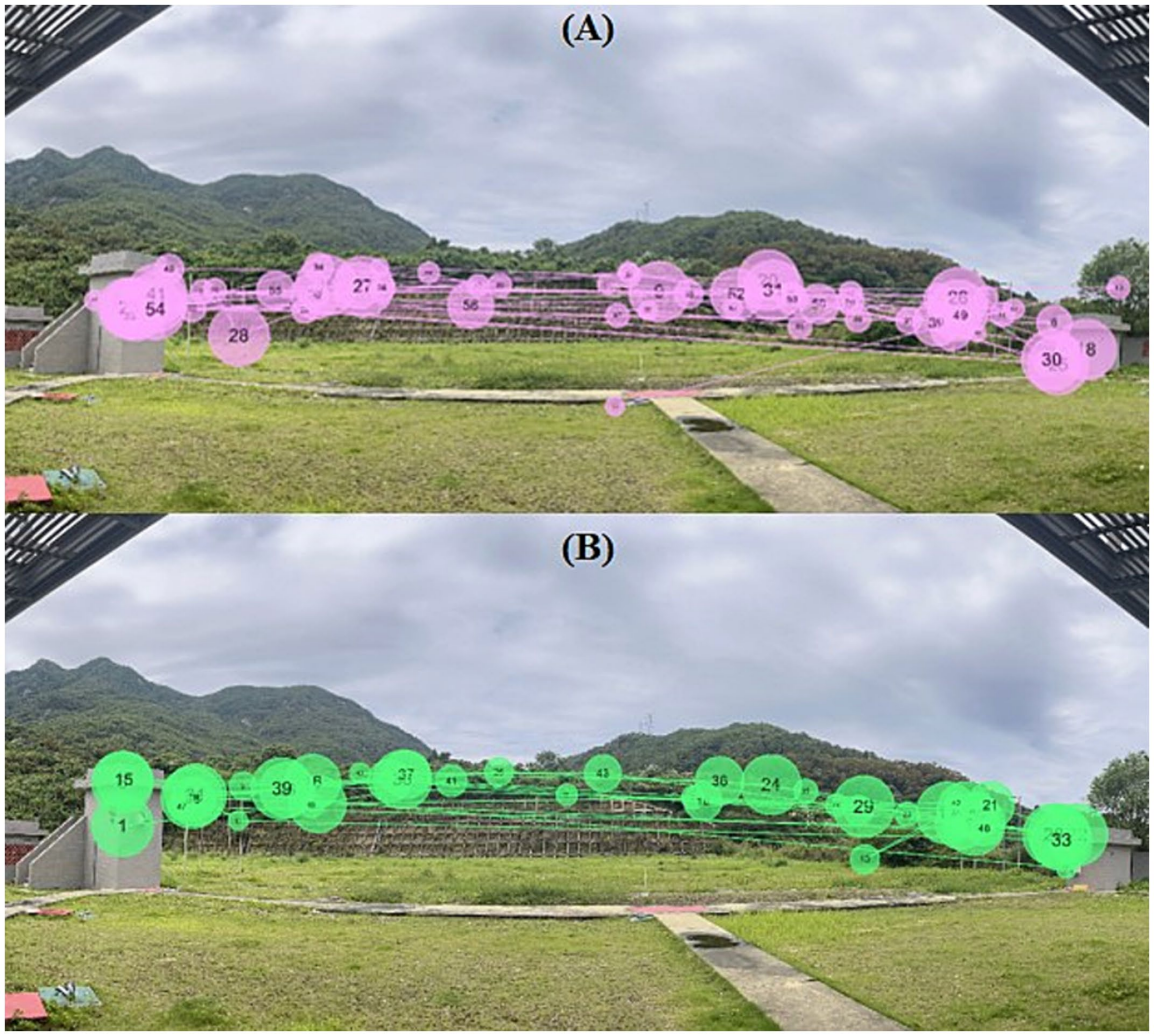
Representative gaze trajectories of novice **(A)** and expert shooters **(B)** during target viewing. Lines represent saccadic eye movements, and circles represent fixation locations. Experts showed more focused and streamlined gaze trajectories compared with novices across target conditions.

As shown in Figure 7, clear differences were observed between expert (Figure 7B) and novice shooters (Figure 7A) in the spatial distribution of gaze trajectories. The expert group exhibited gaze trajectories that were more concentrated within task-relevant regions, with longer and more stable fixations and fewer transitions between fixation points. The fixation sequence was compact and organized, indicating a focused visual search pattern with sustained attention on critical areas of the target trajectory. In contrast, the novice group displayed more dispersed gaze trajectories, characterized by a wider spatial distribution of fixation points and more frequent gaze shifts at multiple regions. Fixations were less consistently located within task-relevant areas, and gaze transitions appeared more frequently, resulting in a less structured trajectory pattern.

All these visual implications are in agreement with the numerical values given in Table 3 (e.g., fixation count: 6.86 ± 0.30 vs. 8.11 ± 0.41 in high house double-target trials; fixation duration: 1.38 ± 0.13 s vs. 1.20 ± 0.05 s). These differences suggest variation in how visual information was sampled and organized during target viewing between the two groups.

## 4 Discussion

The current experiment showed that there were significant variations in eye-movement patterns and visual search features between the expert and novice skeet shooters who were assessed under simulated target-viewing conditions. Overall, professional shooters displayed more visual stability and focus, with a reduced number of blinks, longer fixation periods, smaller magnitude of saccades, and faster behavioral reaction. These characteristics reveal a high degree of perceptual efficiency and task recognition capability, which can be trained over a long period of time and in response to competitive experience. These results show that visual search strategies during target tracking vary systematically across the different levels of performance, which illustrates the influence of experience in defining temporal, spatial, cognitive, and behavioral dimensions of processing visual information. It should be noted that the present experimental paradigm primarily assessed visual detection and anticipatory tracking during simulated target viewing rather than the complete shooting performance sequence. Therefore, the findings should be interpreted as reflecting perceptual–visual components of skill, particularly visual information acquisition and gaze stabilization, rather than direct measures of shooting accuracy or motor execution. While these perceptual processes are fundamental to successful shooting, future studies incorporating behavioral performance measures (e.g., shot accuracy, gun movement dynamics) would further clarify the relationship between visual search behavior and actual shooting performance.

In this study, from a temporal perspective, expert shooters showed fewer fixations as well as longer fixation durations than novices. The trend indicates that professionals can retrieve information related to the task more effectively and can be sustainable in achieving attention to important visual areas without the need to change gaze frequently. The efficiency associated with information processing could be improved with the help of long-term professional training that enables specialists to recognize the main peculiarities of the target path rapidly and minimize the amount of eye movements that are not necessary to follow (Guo Y. et al., 2024; Nascimento et al., 2021). Their gaze behavior indicates a stable target-locking strategy, which enables them to continuously track of the disc target with minimal visual adjustment. Conversely, inexperienced shooters who have little experience are also more vulnerable to external distractions and need more regular fixations to retrieve task-relevant information again (Guo et al., 2025). In skeet shooting, effective target viewing is vital, as it directly influences gun initiation, gun movement speed, and shooting accuracy. Continuous observation of the target makes expert shooters acquire a low-frequency, steady gaze pattern that facilitates accurate visuomotor coordination (Guo Y. et al., 2024).

The present findings are also consistent with previous studies conducted on other sports. In a study carried out on football players, it has been observed that expert athletes had fewer fixations and more efficient visual search behavior than novices (Roca et al., 2018). Likewise, it can be seen that advanced basketball players exhibited a reduced reaction time, fewer fixation points, increased fixation periods, and selective attention to target areas (Jin et al., 2023). The same trends were observed in tennis players, with professional players exhibiting reduced reaction-time, narrowed pupil, and increased fixations on task-related areas, which is in favor of efficient predictive processing (Lin et al., 2021). In badminton sports, expert athletes also displayed a significant difference in gaze timing and allocation during critical phases of play compared with novices (Guo L. et al., 2024). All these findings collectively indicate that the highly trained athletes are using fine-tuned visual search methods that focus on the critical information by reducing unwarranted visual search.

Spatial characteristics of visual search also differed significantly between these two groups. The current study has established that professional skeet shooters had reduced saccade amplitudes compared to novices, which means that they narrow visual scan to locate the target being tracked. Skeet shooting is a fast response exercise that involves relatively simple visual scenes (Nascimento et al., 2021). Experts appear to rely on efficient information-integration mechanisms that might enable them to yield meaningful cues with small, controlled saccades (Guo Y. et al., 2024). Their visual search strategy is guided by anticipation of target flight characteristics. This enables precise target locking and continuous tracking with minimal spatial dispersion (Gao D. et al., 2025). New shooters, on the contrary, had increased saccade sizes indicating a more diffuse and less orderly search strategy due to uncertainty and lack of experience (Guo Y. et al., 2024; Nascimento et al., 2021).

It is also important to consider the potential influence of ocular dominance and handedness on visual tracking under different target flight directions. In the present study, all participants were right-handed and right-eye dominant, which reflects the typical alignment used in skeet shooting. High-house and low-house targets travel in opposite spatial directions, potentially engaging different regions of the visual field and hemispheric processing. Although dominant eye and handedness were not included as independent factors due to homogeneous distribution across groups, their interaction with target flight direction may influence gaze stability and spatial tracking efficiency. Future studies using asymmetric participant samples or binocular gaze analysis may further clarify how ocular dominance contributes to visual search behavior during directional target tracking.

Other games have also been reported to have similar findings. In one study, players in football have been found to have focused fixation patterns and reduced saccades in critical decision-making situations (Gao T. et al., 2025). The same visual search properties have been noted in karate competitors, in which novices showed lower sensitivity to relevant stimuli and less efficient information retrieval (Bandow and Witte, 2020). In a study of race walking referees, it was also found that the expert strategies employed visual searches that were more rapid and accurate, and were marked by reduced saccades and shorter gaze durations (Huijing and Yang, 2024). Since, as long-term training allows experts to achieve stable and effective eye-movement patterns (Lochhead et al., 2024), this can aid in the accurate positioning of the disc target by expert skeet shooters. In contrast, novices often require broader visual exploration to compensate for less developed perceptual templates (Silva et al., 2022). Conversely, coaching interventions have been demonstrated to enhance visual attention and performance with novice athletes but have no significant impact on experts, who already have developed mature search strategies (Ben Chikha et al., 2024). Combined with cumulative sports practice, accumulated experience enables elite athletes to have a sustained fixation in the functional target-viewing areas, creating a stable visual reference frame to predict the target trajectory and to direct them in subsequent actions (Espinosa et al., 2023; Mann et al., 2007; Engel et al., 2022).

It should also be noted that eye-movement data in the present study were recorded at a sampling rate of 60 Hz using a mobile eye-tracking system. While higher sampling frequencies can provide more precise temporal characterization of rapid eye movements, including microsaccades and very short saccadic events, a 60 Hz sampling rate is commonly used in mobile eye-tracking studies examining visual search behavior in applied sports environments. Because the primary focus of the present study was on broader visual search indicators such as fixation duration, fixation count, mean saccade amplitude, and gaze distribution patterns, the sampling rate was sufficient to capture the relevant characteristics of visual search behavior during the simulated target-viewing task. Nevertheless, future studies employing higher-frequency eye-tracking systems may allow more detailed analysis of fine-grained saccadic dynamics during rapid target tracking. The variation in cognitive and behavioral indicators further demonstrate differences in expertise-based benefits in visual processing (Mann et al., 2007). The pupil diameter of expert shooters and the number of blinks were smaller than those of novices, and this may indicate reduced cognitive load and an increased level of attentional stability when viewing the target. Minimal blinking reduces visual distraction at the critical points and aids in constant reception of information, which is important in time-limited activities like skeet shooting (Guo Y. et al., 2024). Pupil diameter is closely linked to cognitive effort and neural activation, particularly within prefrontal regions involved in attentional control (İşbilir et al., 2019; Alnæs et al., 2014; Joshi et al., 2016). The alterations in the pupil size have been identified to indicate the modulation of neural processing based on the task requirements and sensory input (Zhang et al., 2023; Siefert et al., 2024). In visually demanding sports, expert athletes are thought to require fewer cognitive resources to process task-relevant information, reflecting a more efficient perception–action link (Espino Palma et al., 2023). In contrast, novices usually allocate more mental resources to visual processing, which causes neural activity and enlarged pupil diameters (Eldar et al., 2013).

The current results align with the past studies, which indicated that expert athletes had a smaller pupil diameter and a lesser prefrontal stimulation than novices (Keshvari et al., 2024). Keshvari et al. (2024) observed smaller pupil changes and smoother performance in expert participants, indicating reduced cognitive effort. This has been observed in volleyball players, where expert volleyball players were found to have a low pupil diameter and less activation of the prefrontal cortex compared to novice volleyball players, indicating that expert players have more efficient neural processing when performing a task (Gao et al., 2024). These results indicate that expertise may be associated with optimized neural and perceptual mechanisms that reduce cognitive load during visual tasks. Behavioral indicators provide further support to this interpretation. Experts showed high-speed reaction time compared to beginners, which indicated faster information-processing speed, better predictive capacity, and more automation of the perception-action relationship (Guo Y. et al., 2024; Nascimento et al., 2021; Gao D. et al., 2025). Long-term training enables experts to rapidly recognize target patterns and initiate appropriate motor responses with minimal deliberation (Guo Y. et al., 2024). This automation allows visual information to be translated into action more efficiently, contributing to superior performance under time pressure (Guo and Wang, 2025).

The above-mentioned visual search characteristics are consistent with findings in other sports and align with the experience advantage hypothesis, which proposes that expertise is rooted in superior information-processing strategies developed through extensive training (Silva et al., 2022; Guo et al., 2025; Mann et al., 2007). The results provide a basis for designing targeted visual training interventions for novice shooters, with the aim of improving gaze stability, tracking efficiency, and overall visual search performance.

## 5 Conclusion

This study demonstrated significant differences in visual search patterns between expert and novice skeet shooters across simulated target-viewing tasks. During target viewing, expert athletes exhibited longer fixation durations, fewer fixations, smaller saccade amplitudes, reduced blink counts, smaller pupil diameters, and more concentrated and regular eye-movement trajectories. These characteristics indicate more efficient visual information acquisition and stable visuomotor control in expert shooters. Although these findings share some similarities with the concept of the quiet eye, the present task did not include a discrete movement execution phase; therefore, the results should be interpreted as reflecting general gaze stability rather than a formally defined quiet eye period.

Although visual attention heat maps and gaze trajectories suggested group-level differences, these visualizations were primarily qualitative. Because the eye-tracking system provides continuous gaze mapping relative to the dynamic visual scene, future studies may benefit from incorporating quantitative gaze–target coupling measures, such as gaze–target distance, pursuit gain, and temporal alignment between gaze and target motion. Such analyses would provide a more precise characterization of how visual tracking behavior is synchronized with moving targets and may further clarify expertise-related differences in visuomotor coordination.

Although the simulated target-viewing paradigm could not fully replicate real shooting performance, the present task primarily assessed perceptual–visual components of skill, particularly visual detection, anticipatory tracking, and gaze stabilization. The relatively small sample size limits statistical power and the precision of effect estimates, particularly given multiple outcome measures and task conditions. This limitation partly reflects the restricted availability of highly trained skeet shooters at the elite level. In addition, the technical characteristics of the mobile eye-tracking system, including the 60 Hz sampling frequency, may limit the detection of very fine-grained eye-movement events such as microsaccades or extremely rapid saccadic dynamics. In addition, several of the observed effect sizes were relatively large, which may partly reflect sampling variability associated with small group sizes; therefore, the magnitude of these effects should be interpreted with caution. Consequently, null findings and marginal patterns should also be interpreted cautiously, and replication with larger samples would be valuable to confirm the robustness and generalizability of the observed effects.

The present findings nevertheless provide useful insights into expertise-related visual search behavior in skeet shooting. Future research may apply these results to the development of targeted visual training programs, and expert visual search models could be used to guide visual intervention strategies, including video-based feedback, to support performance improvement in developing athletes.

## Data Availability

The raw data supporting the conclusions of this article will be made available by the authors, without undue reservation.
